# Application of Recombinant Proteins for Serodiagnosis of Visceral Leishmaniasis in Humans and Dogs

**DOI:** 10.7508/ibj.2016.03.001

**Published:** 2016-07

**Authors:** Mahin Farahmand, Hossein Nahrevanian

**Affiliations:** Department of Parasitology, Pasteur Institute of Iran, Tehran, Iran

**Keywords:** Visceral leishmaniasis, Recombinant proteins, Diagnosis

## Abstract

Visceral leishmaniasis (VL) is a zoonotic disease caused by *leishmania* species. Dogs are considered to be the main reservoir of VL. A number of methods and antigen-based assays are used for the diagnosis of leishmaniasis. However, currently available methods are mainly based on direct examination of tissues for the presence of parasites, which is highly invasive. A variety of serological tests are commonly applied for VL diagnosis, including indirect fluorescence antibody test, enzyme-linked immunosorbent assay (ELISA), dot-ELISA, direct agglutination test, Western-blotting, and immunochromatographic test. However, when soluble antigens are used, serological tests are less specific due to cross-reactivity with other parasitic diseases. Several studies have attempted to replace soluble antigens with recombinant proteins to improve the sensitivity and the specificity of the immunodiagnostic tests. Major technological advances in recombinant antigens as reagents for the serological diagnosis of VL have led to high sensitivity and specificity of these serological tests. A great number of recombinant proteins have been shown to be effective for the diagnosis of leishmania infection in dogs, the major reservoir of *L. infantum*. Although few recombinant proteins with high efficacy provide reasonable results for the diagnosis of human and canine VL, more optimization is still needed for the appropriate antigens to provide high-throughput performance. This review aims to explore the application of different recombinant proteins for the serodiagnosis of VL in humans and dogs.

## INTRODUCTION

Visceral leishmaniasis (VL), or Kala-Azar, is a zoonotic disease transmitted by female sand flies and caused by *leishmania* species. The parasites are transmitted by the bite of female phlebotomine sand flies to the vertebrate host, where the infecting promastigotes differentiate into amastigotes; this is fatal if left untreated. Leishmania species causing VL have been shown to be capable of infecting humans as well as domestic and wild animals in the Old and New Worlds^[^^1^^,^^2^^]^. VL is endemic in northwestern and southern parts of Iran with about 100–300 new cases reported annually. Domestic dogs (*Canis familiaris*) are considered to be the main reservoir host^[^^3^^-^^6^^]^, some of which are asymptomatic, while others represent clinical signs ranging from mild popular skin diseases to sever forms characterized by cachexia, alopecia, keratoconjunctivitis, anorexia, weight loss, and death^[^^7^^-^^9^^]^. A close contact between dogs and human populations increases the risk of parasite transmission from dogs to sand flies and then to humans^[^^10^^]^. Human VL is the most severe form of the disease characterized by prolonged fever, splenomegaly, hepatomegaly, substantial weight loss, progressive anemia, pancytopenia, and hyper gammaglobulinemia. Human VL is nearly always fatal^[^^11^^]^, unless treated; therefore, early diagnosis of human VL is essential for prevention of mortality and morbidity. A variety of methods have been currently used for the diagnosis of VL, based on aspirates or biopsy specimens of tissues (such as spleen, liver, and bone marrow), in which bone marrow samples display less specificity while spleen samples exhibit more specificity^[^^9^^,^^11^^]^. The specimens are subjected to microscopic examination and cultured on Novy-McNeal-Nicolle and RPMI-1640 media supplemented with 20% fetal calf serum, 2 mM L-glutamine, 55 μg/ml penicillin and 125 μg/ml streptomycin^[^^12^^]^. The growth of promastigotes typically takes between 2-14 days. 

Due to the high risk of bleeding in splenic aspiration, serological assays are used for the detection of antibodies in serum samples derived from humans and dogs. Indirect fluorescent antibody (IFA), indirect hemagglutination assay, complement fixation, direct agglutination test (DAT) and enzyme-linked immunosorbent assay (ELISA) are some of these assays with different specificities and sensitivities^[^^13^^-^^15^^]^. In these tests, antigens are derived from either whole parasite or total parasite lysate. However, development of a recombinant Leishmania antigen would be a valuable platform for serological diagnosis because the production of such an antigen would be parasite growth-independent and more standardized^[^^16^^]^. This review seeks to survey the application of different recombinant proteins for the serodiagnosis of VL in both humans and dogs. 

**Recombinant proteins for ELISA**

ELISA is a serological test used to diagnose the majority of infectious diseases including leishmaniasis. Although the technique is sensitive, its specificity depends on the applied antigen. Several antigens have been already used for immunodiagnostic methods but most commonly is crude soluble antigen (CSA)^[^^2^^,^^5^^,^^11^^,^^13^^]^. To prepare CSA, promastigotes of *L. infantum *are disrupted by ultrasonic treatment, and the supernatant will be used as a soluble antigen to coat ELISA plates^[^^11^^]^. The CSA ELISA shows a considerable overlapping in antibody titers between the controls and patients with active VL. On the other hand, the application of CSA as a diagnostic antigen can suffer from cross-reactivity with other diseases, such as leprosy, malaria, and tuberculosis, where these diseases are endemic^[^^17^^,^^18^^]^. To this end, emphasis has been focused on leishmania antigenic components so that recombinant antigens have been evaluated to be substituted with native parasite antigens, including rkE16 (recombinant kE16), rk26, rK39, and rA2 for serological tests^[^^15^^,^^19^^, ^^20^^]^.

rK39, an antigen for VL diagnosis, exhibits a repetitive immune-dominant epitope in Kinesin-related proteins and is highly conserved among VL species^[^^21^^]^. Several studies carried out in VL-endemic areas have demonstrated that the rK39 ELISA is a sensitive and a specific method for the serodiagnosis and prognosis of human VL^[^^21^^-^^26^^]^. Additionally, preliminary studies have shown that the rK39 ELISA test is most amenable for the serodiagnosis of canine leishmaniasis^[^^27^^]^. In a study performed by Taran *et al*.^ [^^28^^]^, rK39 dipstick test was used to evaluate the performance of K39 sub recombinant antigen from *L. infantum* LON49, which its sensitivity and specificity were found to be 90.7% and 95.6%, respectively. Results from their study suggested that rK26 is a specific antigen for the detection of antibodies in sera from patients with VL infection^[^^29^^]^. In addition, Farajnia *et al*.^[^^30^^]^ evaluated the performance of rK26 by ELISA and demonstrated that the sensitivity of this antigen among VL-confirmed patients was 96.8%. Pattabhi *et al.*^[^^31^^]^ produced rK28 and used it in ELISA as an antigen. They also evaluated VL-confirmed sera isolated from Sudanese patients and observed the sensitivity and specificity of 96.8% and 96.2%, respectively. KE16 from *L. donovani* is another *kinesin* gene derived from leishmania species that has been successfully cloned and expressed. The rKE16 was used^[^^1^^,^^9^^]^ in ELISA to evaluate the serodiagnostic capacity of Indian Kala-Azar^[^^32^^,^^33^^]^. Results from those studies showed that the expressed Ld-rKE16 antigen can serve as a highly specific and sensitive tool for VL diagnosis^[^^32^^,^^33^^]^. 

KMP11, rH2A and the Q protein are the antigens expressed in *L. donovani* complex (*L. donovani*,* L. infantum syn. *and *L. chagasi*). The recombinant antigen “Q” protein is a chimeric protein formed by the genetic fusion of five antigenic determinants from four *Leishmania *proteins. The fragments forming Q antigen are highly immunogenic during *L. infantum* infection. In a study, when KMP11, rH2A, and the Q protein were used as antigens in ELISA for the diagnosis VL patients, the sensitivity of ELISA was demonstrated to be high^[^^34^^]^. The A2 proteins of *L. donovani* are a stage-specific protein present in the amastigote stage, appearing to facilitate visceralization of parasites in the mammalian host. In *L. donovani*, these proteins range in size from 42 to 100 kDa and have highly conserved repetitive elements located on chromosome 22 in tandem arrays^[^^35^^-^^38^^]^. In a previous study, we demonstrated that A2 proteins are present in amastigote form of *L. infantum* isolated from dogs in Meshkin-Shahr, Ardebil Province, North-Western Iran^[38]^. It is well-established that the A2 protein is overexpressed in the amastigote stage in *L. donovani* as well as *L*. *mexicana *species complex, including *L. amazonensis*, but not in *L*. *tropica *or *L*. *braziliensis *species complexes. At the same time, Anti-A2 antibodies were detected in human and dog sera suffering from active VL^[^^36^^,^^39^^]^. In a study carried out by Porrozzi *et al.*^[^^17^^]^, ELISA was used for serodiagnosis of symptomatic and asymptomatic VL in dogs based on crude and recombinant proteins (rA2, rK26, and rK39). In addition, it was found that rA2 is more sensitive to asymptomatic dogs, when compared to the soluble antigens, rK39, and rK26. 

In a study performed by Souza *et al.*^[^^40^^]^, the recombinant antigen, ATP diphosphohydrolase (rLic-NTPDase-2), was produced and validated for use in the immunodiagnosis of VL. In brief, a microplate was coated with the purified rLic-NTPDase-2 antigen for the ELISA assay. Next, serum samples from naturally infected dogs in Brazil were tested for VL detection. Although showing high sensitivity and specificity (100%), this test was not able to distinguish VL. Therefore, it seems that rLic-NTPDase-2 may not be a useful marker for VL prognosis. 

ELISA-rHsp83 was used as an antigen to detect anti-leishmania antibodies, which showed significantly high sensitivity^[^^16^^]^. In a study performed by Boarino *et al*.^[^^41^^]^, a combination of K9, K26, and K39 sub recombinant chimeric antigens was coated in a single well and used to diagnose human and canine VL by an indirect ELISA assay. A total of 384 human and 609 canine serum samples were tested, in which the specificity was found to be suitable for both human and canine samples (99%) but the sensitivity of chimeric ELISA was higher in canine VL (96%) than in human VL (82%).

To determine antibodies in the control and VL patients in Sudan, an immunodominant kinesin protein (rKLo8) from an autochthonous *L. donovani* strain was expressed, used as an ELISA coating antigen, and compared with cloned kinesin proteins of *L. infantum* (synonymous *L. chagasi*) (K39) and *L. donovani* (KE16). Among 106 parasitologically VL-confirmed patient sera, the sensitivity and specificity of rKLo8-coated ELISA were 98.1% and 96.1%, respectively, as compared to 96.2% sensitivity of rk39^[^^42^^]^. In a study done by Islam *et al.*^[^^43^^]^, rKRP42, the recombinant kinesin-related protein of *L. donovani*, with a molecular weight of 42 kDa, was produced and evaluated in urine-based ELISA test for the diagnosis of VL. The rKRP42 urine ELISA showed a high sensitivity of 94% and specificity of 99.6% among VL samples. 

More recently, two novel recombinant antigens, the multiepitope proteins PQ10 and PQ20, were used in canine VL ELISA, which were able to identify asymptomatic dogs (80%). PQ10 consists of a 10-codon sequence present in antigenic peptides, while PQ20 includes a 20-codon sequence exist in T- and B-cell epitopes, respectively. In addition, a flexible linker was used as a spacer between epitope sequences. These antigens was used for VL ELISA and demonstrated to be sensitive enough for the identification of asymptomatic dogs (80%)^[^^44^^]^. 

**Recombinant proteins for the r** **apid immune-chromatographic dipstick test** 

The rapid immunochromatographic dipstick test is a qualitative test able to detect anti-*Leishmania* circulating antibodies by the leishmanial recombinant antigen, such as rK39 and is adapted for use under field conditions. The rK39 antigen is the product of a gene cloned from *L. chagasi *containing a 39-amino-acid repeat. Taken together, K39 is a part of a 230-kDa protein that is encoded by a kinesin-like gene of *L. chagasi* and conserved among viscero-tropic *Leishmania *species^[^^19^^,^^43^^]^. The rapid rK39 immuno-chromatic dipstick test is both sensitive (67-100%) and specific (70-100%)^[^^42^^,^^45^^-^^49^^]^. However, the cross-reactivity with malaria, enteric fever, disseminated tuberculosis and *Toxoplasma gondii* may occur*.* In a study carried out in Iran, the sensitivity and specificity of the rK39 strip test were compared with the immunofluorescent antibody test in 47 children suspected to VL. The sensitivity and specificity of the strip test were 82.4% and 100%, while those of the immunofluorescent antibody test were 100% and 92.7%, respectively^[^^50^^]^. Mohebali *et al*.^[^^51^^]^ compared the rK39 dipstick test with DAT to detect *L. infantum* infection in dogs in endemic areas in Iran. The tests were carried out on 116 clinically suspected dogs and 152 healthy controls from endemic areas of Ardabil Province, North-Western Iran. A sensitivity of 70.9% and specificity of 84.9% were found for the rK39 dipstick test.

In a literature search for VL detection, 31 studies have used dipstick form of rK39 in dogs. Their results revealed that the overall sensitivity was 86.7% in symptomatic dogs. The reasons for such variability are not clear; in addition, the number of studies for such analysis is too small for a detailed comparison of different tests and geographical areas^[^^47^^]^. In parallel, a clinical cohort study in Sudan was designed to evaluate the rK28-based RDT for diagnosis of *L. donovani* VL. The serum analysis of VL-suspected individuals possesses a reasonable specificity and sensitivity of 97.6% and of 94.5%^[^^53^^]^. 

The rkE16 dipstick test is a one-step rapid immunochromatographic test for diagnosis of Kala-Azar. The test is based on a 39-amino-acid protein, the rKE16 antigen, derived from the C-terminus of the kinesin protein from an Indian isolate of *L. donovani*^[^^32^^]^. In our previous study, the average of positive cases among asymptomatic dogs was 32.4% with rKE16^[^^9^^]^. 

**Recombinant proteins for the l** **atex agglutination test**

In the majority of immunoagglutination assays, latex is the main particle for antigen absorption. The attachment of molecules to latex particles can be achieved through either physical adsorption or covalent coupling. For visual slide agglutination, a particle diameter range of 0.2–0.9 mm is needed^[^^54^^]^. The only recombinant antigen used for the latex test is rk39, which is generally used in latex agglutination at a concentration of 1 mg/ml in glycine-buffered saline. The sensitivity of the latex test among parasitologically proven index cases was only 80%, which was due to a lower power of antibody detection by this test^[^^55^^]^. 

**Recombinant proteins for other serological assays**

Several serological tests are used for the diagnosis of VL, including IFA, DAT, ELISA, and dot-ELISA. The mentioned tests are mostly based on purified, water-soluble antigen fractions of promastigote or amastigote stages or on recombinant antigens. It is notable that recombinant proteins are only used in ELISA, latex agglutination test, and rapid immunochromatographic dipstick test, but not in other assays. On the basis of publications, there are no report indicating that recombinant antigens can be used for IFA and DAT assays. 

## Conclusion

The technological advances in recombinant antigens as reagents for the serological diagnosis of VL have resulted in high sensitivity and specificity of the serological tests. Several recombinant proteins have been shown to be useful for the diagnosis of leishmania infection in dogs. It should be noted that dogs are considered as a major reservoir of *L. infantum *and, therefore, promote the spread of human leishmaniasis. Several proteins able to generate substantial titers of antibodies and are detectable in canine VL, including KMP11, LiP, rk39, rk26, rA2, rk9, rKE16, and histones such as H_2_A. Early diagnosis and control of canine VL can reduce the transmission of infection from asymptomatic dogs to sand flies and humans. Different types of antigens with variable sensitivity and specificity have been used in ELISA and in rapid immunochromatographic dipstick tests, depending on both the kind of recombinant antigens and the areas under study. 

ELISA is a sensitive and specific test for the detection of antibodies in humans and dogs infected with VL. Anti-A2 antibodies are easily detected by a rA2 protein containing a tag of six histidine residues (A2-HIS), in 77% of the patient sera with symptomatic VL and in 87% of the sera from dogs, which were positive for *Leishmania *immunofluorescent antibody test (IFAT) or parasitological evaluation. Anti-A2 antibodies have been also detected in symptomatic and asymptomatic dogs^[^^17^^,^^20^^,^^41^^] ^.

Data from such studies have indicated that using a combination of recombinant proteins in a single-well test would be better for diagnosis of human and canine VL by an indirect ELISA. The rapid immunochromato-graphic dipstick test, for example rK39 strip test, is a simple and cost-effective assay that uses chromate-graphic strips impregnated with the rK39 antigen and can be easily used in the field. Several studies have shown that the rK39 strip test is a reliable test for VL diagnosis with no false positives. In comparison to DAT and ELISA, dipstick rK39 is a simple, more practical, sufficiently sensitive and highly specific method for the diagnosis of active VL in humans as well as in dogs. Furthermore, the test can be performed with a few drops of blood, serum, or plasma in field conditions^[^^2^^]^. 

There are several serological tests used for the diagnosis of VL in humans and dogs. Moreover, reasonable levels of sensitivity and specificity are particularly desirable to avoid false negative and false positive reactions. This issue can lead to disease transmission and unnecessary euthanasia of healthy dogs. The problems with the conventional assays (such as IFAT, DAT, and ELISA) may be overcome by using recombinant polypeptides containing a specific epitope that is able to elicit an immune response in the majority of dogs and humans with VL. Results indicate that each antigen carries immunodominant epitopes, the combination of which may further increase the sensitivity of currently available serological tests^[^^17^^]^. Although several recombinant proteins with high efficacy end up with reasonable results for the diagnosis of human and canine VL, more optimization is still required for appropriate antigens to provide high-throughput performance.
